# An Ensemble Spectral Prediction (ESP) model for metabolite annotation

**DOI:** 10.1093/bioinformatics/btae490

**Published:** 2024-08-17

**Authors:** Xinmeng Li, Yan Zhou Chen, Apurva Kalia, Hao Zhu, Li-ping Liu, Soha Hassoun

**Affiliations:** Department of Computer Science, Tufts University, Medford, MA, 02155, United States; Department of Computer Science, Tufts University, Medford, MA, 02155, United States; Department of Computer Science, Tufts University, Medford, MA, 02155, United States; Department of Computer Science, Tufts University, Medford, MA, 02155, United States; Department of Computer Science, Tufts University, Medford, MA, 02155, United States; Department of Computer Science, Tufts University, Medford, MA, 02155, United States; Department of Chemical and Biological Engineering, Tufts University, Medford, MA, 02155, United States

## Abstract

**Motivation:**

A key challenge in metabolomics is annotating measured spectra from a biological sample with chemical identities. Currently, only a small fraction of measurements can be assigned identities. Two complementary computational approaches have emerged to address the annotation problem: mapping candidate molecules to spectra, and mapping query spectra to molecular candidates. In essence, the candidate molecule with the spectrum that best explains the query spectrum is recommended as the target molecule. Despite candidate ranking being fundamental in both approaches, limited prior works incorporated *rank learning* tasks in determining the target molecule.

**Results:**

We propose a novel machine learning model, Ensemble Spectral Prediction (ESP), for metabolite annotation. ESP takes advantage of prior neural network-based annotation models that utilize multilayer perceptron (MLP) networks and Graph Neural Networks (GNNs). Based on the ranking results of the MLP- and GNN-based models, ESP learns a weighting for the outputs of MLP and GNN spectral predictors to generate a spectral prediction for a query molecule. Importantly, training data is stratified by molecular formula to provide candidate sets during model training. Further, baseline MLP and GNN models are enhanced by considering peak dependencies through label mixing and multi-tasking on spectral topic distributions. When trained on the NIST 2020 dataset and evaluated on the relevant candidate sets from PubChem, ESP improves *average rank* by 23.7% and 37.2% over the MLP and GNN baselines, respectively, demonstrating performance gain over state-of-the-art neural network approaches. However, MLP approaches remain strong contenders when considering top five ranks. Importantly, we show that annotation performance is dependent on the training dataset, the number of molecules in the candidate set and candidate similarity to the target molecule.

**Availability and implementation:**

The ESP code, a trained model, and a Jupyter notebook that guide users on using the ESP tool is available at https://github.com/HassounLab/ESP.

## 1 Introduction

Nontargeted tandem mass spectrometry is a powerful approach to characterize small molecules produced in cells, tissues, and other biological systems. Unlike genomics that specify the cell’s capabilities, metabolites are direct products of enzymatic reactions and provide an accurate functional readout of cellular state (Baker 2011, Patti *et al.* 2012). So far, metabolomics studies have identified disease biomarkers, elucidated biochemical changes associated with drug responses, created opportunities for personalized medicine, and analyzed relationships between diet and health (Kitano 2002, Johnson *et al.* 2016, Chong *et al.* 2018, Jacob *et al.* 2019). Importantly, the ability to collect thousands of measurements on the sample-under-study promises to broadly profile the metabolome and revolutionize phenotyping and advancing biological discovery.

A key challenge in metabolomics is the “annotation” problem, where measured spectra are assigned chemical identities. Currently, only a small fraction of measured spectra is annotated (da Silva *et al.* 2015). As spectral libraries are limited and experimental exploration is costly and time-consuming, computational approaches have emerged as an effective complementary alternative (Liebal *et al.* 2020). The ability to translate between molecular and spectral views has given rise to spectrum-to-molecule and molecule-to-spectrum approaches. In the spectrum-to-molecule approach, a query spectra is mapped to a molecular structure or properties. CSI:FingerID (Dührkop *et al.* 2015) predicts molecular fingerprints for a set of candidate molecules, and then ranks the query spectra against the predictions. For each such fingerprint, CSI:FingerID trains Support Vector Machines (SVMs) that utilize kernel similarities to distinguish compounds based on the relevant property. CSI:FingerID fingerprints were further utilized for *de novo* molecular generation (Stravs *et al.* 2022). MassGenie (Shrivastava *et al.* 2021) casts the spectrum-to-molecule problem as a translation problem from binned mass spectral peaks to a SMILES string using the deep learning transformer model (Vaswani *et al.* 2017). MassGenie then uses a variational autoencoder to generate *de novo* candidate molecules that are “close” in the chemical space. Recent approaches learn a joint latent space for both molecules and spectra, allowing for semi-supervised training using unpaired data (Kutuzova *et al.* 2021a,b). MIST is a spectra-to-fingerprint transformer-based model that encodes peaks with their chemical formula representation, and it is trained to predict relevant substructure fragments (Goldman *et al.* 2023). Further, MIST explicitly encodes all pairwise neutral loss relationships as inputs to the attention-layer modules. The MIST model is reported to outperform the prior best-in-class tool, CSI:FingerID (Dührkop *et al.* 2015) within SIRIUS (Dührkop *et al.* 2019), which reported the highest performance annotation tool at CASMI2022 (http://www.casmi-contest.org). Further, MIST’s latent space spectral distances were more effective than other tools, including MS2DeepScore (Huber *et al.* 2021b), Spec2Vec (Huber *et al.* 2021a), and cosine distances, in clustering high-similarity compounds, indicating that MIST learns spectral embeddings that are highly correlated with metabolite structure similarity.

In the molecule-to-spectrum approach, spectra is predicted for candidate molecules that match the chemical formula of the measured spectra. The candidate spectra are then ranked against the query spectra based on spectral similarity. The candidate molecule with the highest scoring spectra is then assigned as the identity of the query spectra. Example approaches include MetFrag (Ruttkies *et al.* 2016), CFM-ID (Allen *et al.* 2014, Wang *et al.* 2021), MLP-based NEIMS (Wei *et al.* 2019), our recent Graph Neural Network-based (GNNs) method (Zhu *et al.* 2020), which was the first to suggest using GNNs for spectra prediction, and MassFormer (Young *et al.* 2021), which is trained on positive mode ESI Orbitrap spectra with specific precursor adducts. With the exception of the Input Output Kernel Regression (IOKR) with magnitude preservation, which utilizes PubChem derived candidates set to learn a mapping from spectra to molecule structure ranking using kernel regression, none of the prior works utilize rank-learning in their approaches.

**Figure 1.**
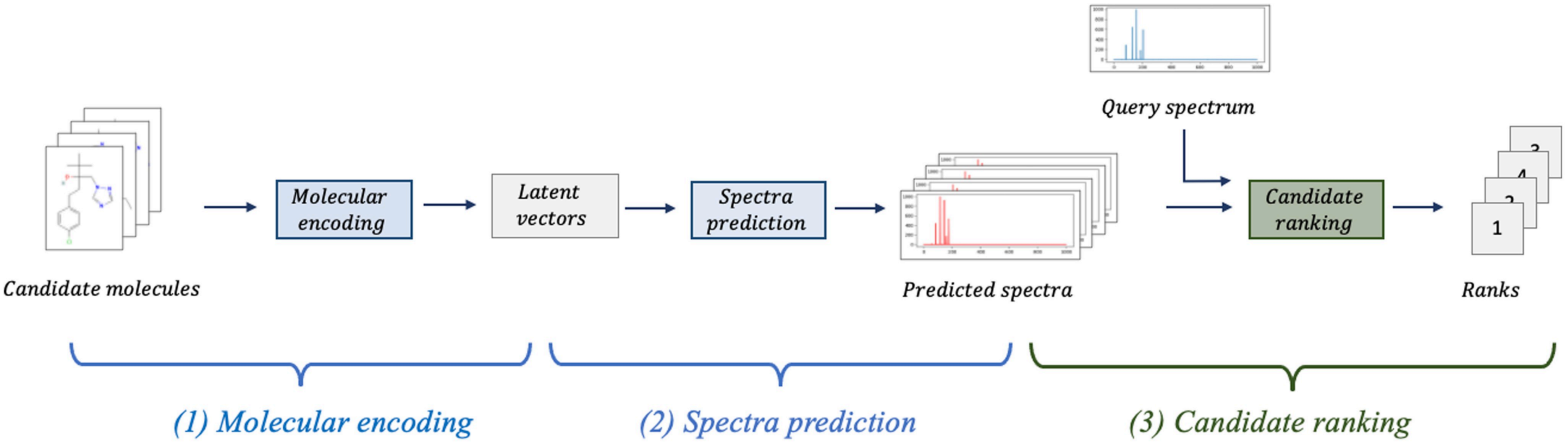
A conceptual framework for solving the three subproblems involved in the molecule-to-spectrum annotation problem: (1) representation learning of molecules, (2) spectra prediction from latent representation vectors, and (3) ranking of candidate spectra against query spectrum using spectral similarity.

We provide in this paper a conceptual framework and an accompanying deep-learning approach for solving the molecule-to-spectrum problem. The problem is conceptually examined as three subproblems (Fig. 1): (i) representation learning of candidate molecules, (ii) mapping candidates to their corresponding spectra, and (iii) learning to rank the predictions of the candidate molecules against the measured spectra. Learning molecular representations, as opposed to fixed fingerprints, allows for flexible representations that may lead to improved top-ranked candidate predictions. Mapping from candidates to spectra is a translation task. Neural network models excel at learning such tasks. Learning to rank candidates however is a challenging task as it implies, at an initial glance, the need for molecular candidate sets and their spectra as training data. Importantly, this conceptual framework can also applied to the spectrum-to-molecule problem (Supplementary Fig. S1), where the ranking can be learned on preselected molecular candidates or on *de novo* candidates.

To implement this framework, we present in this paper a novel molecule-to-spectrum neural network-based ensemble model, referred to as Ensemble Spectral Prediction (ESP). The ensemble model utilizes both MLP- and GNN-based spectral predictions as each can outperform the other for differing molecules. Spectral predictions using MLP and GNN approaches are enhanced by using label-mixing mechanism to capture dependencies among spectral peaks. Multi-tasking on additional data, spectral topic labels obtained using Latent Dirichlet Allocation (LDA) (Blei *et al.* 2003, van Der Hooft *et al.* 2016), is used to further improve MLP and GNN spectral predictions. Importantly, we circumvent solving the challenging problem of retrieving candidate molecules (and their spectra) for our training data. Instead, we partition the molecules in the *training* set based on their chemical formulas and *learn*, based on *ranking* results, how to combine the MLP- and GNN-based spectral predictions to improve spectral prediction. To evaluate our results, we explore two training datasets, NIST-20 (https://chemdata.nist.gov) and a dataset provided with the CANOPUS tool (Dührkop *et al.* 2021) that contains spectra from GNPS (Wang *et al.* 2016) and MoNA (https://mona.fiehnlab.ucdavis.edu) [hereafter called NPLIB1 as per the MIST (Goldman *et al.* 2023) GitHub site], and retrieve candidate molecules from PubChem. We explore various data splits while assuming that the target molecules is in the candidate set. We use random splits, as common in machine learning models, to ensure that the test set distribution reflects that of the training set, and realistic splits that may mirror the chemical novelty of real case use. Further, we address the open question of the impact of the quality of the candidate set (in terms of similarity to the target molecule) on annotation performance. We compare our results with MIST (Goldman *et al.* 2023). Importantly, we show that candidate similarity to the target is a pressing challenge for annotation that is worthy of consideration in future annotation benchmarking efforts. Measuring performance using *average rank* through the ESP model shows remarkable gain over existing neural-network approaches.

## 2 Methods

### 2.1 MLP- and GNN-based molecular encoding

The MLP-based models encode the Extended Connectivity Fingerprint (ECFP) molecular fingerprint, while the GNN-based models encode a molecular graph. The models therefore learn a latent molecular representation vector, **z**:

(1) $$z=z_{\text{MLP}}\;\text{OR}\;z_{\text{GNN}}$$

where $z_{\text{MLP}}$ and $z_{\text{GNN}}$ will be later defined in (2) and (4), respectively.

We first describe the MLP model. In addition to the fingerprint, the MLP model encodes instrument settings, which specifies a one-hot encoding vector of the precursor type, if targeting more than one precursor type, and a value corresponding to the normalized collision energy. The fingerprint and the instrument settings are encoded by a single-layer fully connected neural network, NN(·), to predict the vector $z_{\text{MLP}}$:

(2) $$z_{\text{MLP}}=\text{MLP}(X)$$

with **X** includes both fingerprint $X_{\text{FP}}$ and instrument setting $X_{\text{IS}}$ as features,

(3) $$\text{MLP}(X)=\text{NN}\left(\text{CONCAT}\left(\text{NN}(X_{\text{FP}}),\;\text{NN}(X_{\text{IS}})\right)\right)$$

The GNN model encodes a molecular graph, G=(V,E), where graph nodes v∈V correspond to atoms, and graph edges (u,v)∈E correspond to bonds. We augment node features with the instrument settings. The GNN latent representation vector $z_{\text{GNN}}$ is defined as:

(4) $$z_{\text{GNN}}=\text{GNN}(G)$$

We use Graph Isomorphism Network with Edge features (GINE) (Hu *et al.* 2019) to encode *G*. Besides GINE, we explored a number of GNNs, including Graph Isomorphism Network (GIN) (Xu *et al.* 2018), Graph Attention Networks (GAT) (Veličković *et al.* 2017), Relational Graph Convolutional Networks (R-GCN) (Schlichtkrull *et al.* 2018), and the Weisfeiler–Lehman network (WLN) (Lei *et al.* 2017), which was utilized in our prior work (Zhu *et al.* 2020). Our experiments showed that GINE outperformed other models, potentially due to GIN’s provably maximum discriminative power among GNNs (Xu *et al.* 2018).

We describe the GINE model with *K* layers. Node features contain atom information, including atom type and atom mass $X_{\text{ATOM}}$, and instrument setting $X_{\text{IS}}$; they are encoded by a single-layer fully connected neural network. The node representations are initialized as $h_{v}^{0}$:

(5) $$h_{v}^{0}=\text{NN}\left(\text{CONCAT}\left(\text{NN}(X_{\text{ATOM}}),\;\text{NN}(X_{\text{IS}})\right)\right)$$

Edge features represent bond types. Edge representation at the *k*th layer $h_{e}^{k}$ is computed from the encoded bond type $X_{e}$ as $h_{e}^{k}=\text{NN}(X_{e})$. Assuming $h_{v}^{k}$ is the representation of node *v*, (4) is defined as:

(6) $$m_{v}^{k}=\sum_{u:\left(u,v\right)\in E}h_{u}^{k-1}+\sum_{e=(u,v)\in E}h_{e}^{k-1}$$

(7) $$h_{v}^{k}=\text{ReLU}\left({\text{NN}}^{k}(m_{v}^{k})\right)$$

(8) $$z_{\text{GNN}}=\text{READOUT}(h_{v}^{K})$$

where $m_{v}^{k}$ is the message for node v∈V, updated from its neighboring nodes (u:(u,v)∈E) and connected edges (e=(u,v)∈E). The $z_{\text{GNN}}$ is the molecular representation obtained from a READOUT function using the last layer’s embedding $h_{v}^{K}$. We follow GINE and instantiate READOUT as mean pooling.

### 2.2 Spectra prediction

Given a molecular encoding **z**, we predict the values of peak intensity at binned *m*/*z* ratios. As we discretize our data, we assume *P* discrete bins, where each bin spans a 1-Da range of *m*/*z* values between 0 and 1000 Da. The predicted spectrum, $\hat{y}$, and mass spectra prediction loss, L, are defined as:

(9) $$\hat{y}=\;\text{PRED}(z)$$

(10) $$L=-\text{cos}(\hat{y},y)$$

where PRED(·) is neural network that predicts spectra from molecular representation **z**, and cos(·,·) is the cosine similarity between the predicted spectra, $\hat{y}$, and the query spectra, **y**. For all PRED(·) functions, we applied a two-layer MLP augmented with bidirectional prediction mode (Wei *et al.* 2019), which increases the prediction accuracy on the larger fragments that arise due to neutral losses.

### 2.3 Modeling peak dependencies

Spectral peaks may co-occur in groups reflecting a particular combination of fragments. Encoding such dependencies among co-occurring peaks is beneficial. With this observation, we enhance MLP and GNN models to *learn* dependencies among peaks using two techniques, label mixing and multi-task learning on predicting LDA topic distributions.

To capture dependencies among peaks, we use “mixing” matrices to capture peak-to-peak co-occurrence. Using *L* such matrices $\left(Q_{l}:l=1,\ldots ,L\right)$, the spectral prediction ${\hat{y}}_{co}$, is therefore defined as:

(11) $${\hat{y}}_{co}=\sum_{l}^{L}\hat{y}Q_{l}\tau_{l},\;l\in \{1,\ldots ,L\}$$

where $Q_{l}$ “mixes” entries in an initial prediction $\hat{y}$ and capture co-occurrences of peaks. Each $Q_{l}$ matrix has size P×P, where *P* is the length of a spectrum vector. Here $\sum_{l}^{L}\tau_{l}=1$, and $\tau_{l}$ is a learnable weight assignment for each *Q_l_*. We include *L* such matrices to capture different types of co-occurrence. Importantly, to reduce the number of learned parameters, we consider the parameterization of each *Q_l_* in a lower dimensional space with a lower dimension matrix $D_{l}$, with dimensions of *P *×* M* with *M *<* P* (Supplementary Fig. S8):

(12) $$Q_{l}=D_{l}D_{l}^{⊤}$$

As Equation (11) is a summation of terms containing $D_{l}$ and $D_{l}^{⊤}$, our label mixing formulation cannot be expressed using a linear layer with a single *Q* matrix.

We combine the spectra prediction (after the label-mixing layer) with the original prediction (prior to the label-mixing layer) as follows:

(13) $${\hat{y}}_{\mathrm{update}}=\;\theta \hat{y}+(1-\theta ){\hat{y}}_{co}$$

where ${\hat{y}}_{\mathrm{update}}$ is the updated spectra and *θ* is a hyperparameter. For the rest of the manuscript, we drop the “updated” notation, and use $\hat{y}$ for the updated predicted spectra.

### 2.4 Predicting LDA topic distributions as a secondary task

To further exploit peak dependencies, we run Latent Dirichlet allocation (LDA) in sklearn to learn spectral motifs. LDA allows modeling the spectra in a lower-dimensional subspace. It has been shown that making predictions in a shared subspace is beneficial for multi-label learning (Ji *et al.* 2008). A spectrum can be viewed as a set of bin labels to be predicted. Predicting topics thus regularizes the network’s latent space. LDA was trained on all spectra within the NIST dataset. LDA assigns each spectra one or more spectral topics. We use a spectra matrix $Y=[y_{1}^{⊤},y_{2}^{⊤},\ldots ,y_{N}^{⊤}]$, where *N* is the number of training data points. The LDA model is learned based on **Y** and it assigns likelihood of topic distributions per spectra as follows:

(14) R=LDA(Y)

where LDA(·) is the learned LDA model, and **R** represents the vertical stacking of topic distributions for each spectra, **r**.

We then predict the distribution of LDA topics for each spectra as an auxiliary task using Multi-task Learning (MTL). We assume the predicted topic distribution from the LDA model as ground truth labels. Learning this distribution improves molecular representation and therefore results in enhanced spectral prediction. Assuming T LDA topics, and topic distribution **r**, we define the prediction function and loss for the auxiliary task as follow:

(15) $$\hat{r}=\;\text{AUX}(z)$$

(16) $$L_{\text{AUX}}=-\sum_{t}^{T}r_{t}\;\text{log}({\hat{r}}_{t})$$

where $r_{t}$ is an entry for topic *t* in **r**, ${\hat{r}}_{t}$ is an entry for topic *t* in the predicted spectral topic distribution $\hat{r}$, and the auxiliary function AUX(·) is implemented using two fully connected layers.

### 2.5 Candidate ranking based on spectra predictions

Spectral prediction is performed using both the MLP-PD and the GNN-PD models on molecules within a candidate set *C*. Given a molecule *t*, and its spectra, $y_{t}$, the spectral prediction loss for *t* and the candidates in *C* is computed by comparing the spectral prediction against $y_{t}$ using cosine similarity:

(17) $$L_{t}=-\text{cos}({\hat{y}}_{t},y_{t})$$

(18) $$L_{c}=-\text{cos}({\hat{y}}_{t},y_{c}),\;c\in C$$

Based on the sorted losses, we compute the MLP- and GNN-based rankings, $\mathrm{Ran}k_{t}^{\text{MLP}}$ and $\mathrm{Ran}k_{t}^{\text{GNN}}$, respectively, corresponding to the rank of *t* among the candidates.

### 2.6 Rank-based ensemble model

To use the MLP- and GNN-based spectra predictions to maximize candidate ranking, we train an ensemble model to learn a weighted sum of the MLP-PD and GNN-PD spectral predictions. As our goal is to perform candidate ranking, the model learns the weighted sum of the two predictions based on ranking results. Several steps are required for training the ensemble scoring model.

First, we group spectra in the training set based on their associated molecular formulae. Each such group provides a molecular candidate set (same molecular formulae, but different molecular arrangements) without the need for retrieving candidates or spectra beyond those available in the training set. Each spectra is treated as query spectra with a known target molecule. All other molecules in the group are considered the candidate set.

Second, for each training spectra, its known target molecule, and its candidate set, $\mathrm{Ran}k^{\text{MLP}}$ and $\mathrm{Ran}k^{\text{GNN}}$ are computed. Based on the ranking of the two models, we define labels for the classifier, $d^{\text{MLP}}$ and $d^{\text{GNN}}$, for each query spectra as follows:

(19) $$d^{\mathrm{MLP}}=\{\begin{array}{cc} 1, & \mathrm{Ran}k^{\text{MLP}}\leq \mathrm{Ran}k^{\text{GNN}}\\ 0, & \text{otherwise} \end{array}$$

(20) $$d^{\text{GNN}}=1-d^{\text{MLP}}$$

A $d^{\text{MLP}}$ true value indicates that the query spectra is best ranked by the MLP-based model. As the training of the ensemble model might benefit differently from each training example, we compute a weighting, *γ_i_*, for each sample point in our training data. A sample is more important if the ranking of one of the models is better than the other model, indicating that the winning model has better discriminative power toward the sample. The difference in ranks between the two models also indicates better discriminative abilities. *γ* is therefore computed based on the symmetric mean absolute percentage error (SMAPE) metric as follows:

(21) $$\gamma =\frac{\left|\mathrm{Ran}k^{\text{GNN}}-\mathrm{Ran}k^{\text{MLP}}\right|}{\mathrm{Ran}k^{\text{GNN}}+\mathrm{Ran}k^{\text{MLP}}}$$

Once we obtain the weighting for each training sample, we can then train an ensemble scoring model to predict the MLP and GNN labels, ${\hat{d}}^{\text{MLP}}$ and ${\hat{d}}^{\text{GNN}}$, under loss $L_{\text{ENS}}$:

(22) $${\hat{d}}^{\text{MLP}}=\;f_{\text{ENS}}(y)$$

(23) $${\hat{d}}^{\text{GNN}}=\;1-{\hat{d}}^{\text{MLP}}$$

(24) $$L_{\text{ENS}}=\sum_{i}^{N}\gamma_{i}\cdot \text{BCE}({\hat{d}}_{i}^{\text{MLP}},d_{i}^{\text{MLP}})$$

where $f_{\text{ENS}}$ is a function that approximates the MLP label, and **y** is the spectra in training set. BCE is the Binary Cross Entropy (BCE) function that compares each of the predicted probabilities to its class label, and *N* is the number of training spectra.

When using the ensemble model during the test phase, the MLP-PD and GNN-PD models predict the spectra for the candidate set. Further, ${\hat{d}}^{\text{MLP}}$ and ${\hat{d}}^{\text{GNN}}$ are computed as above on the query spectra **y**. The predicted spectra is therefore computed as:

(25) $$\hat{y}=\;{\hat{d}}^{\text{MLP}}{\hat{y}}_{\text{MLP}}+{\hat{d}}^{\text{GNN}}{\hat{y}}_{\text{GNN}}$$

Candidate ranking then proceeds to identify the best ranking for the query spectra by calculating the similarity on spectra predictions (discussed in Candidate ranking based on spectra predictions).

## 3 Results and discussion

### 3.1 ESP overview

**Figure 2.**
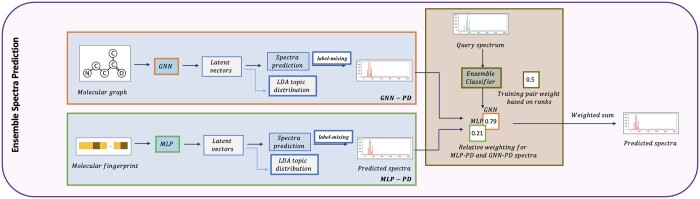
The Ensemble Spectra Prediction (ESP) model has two phases. Phase 1. Molecular encoding using GNN and MLP followed by spectra prediction enhancement using label-mixing to capture spectral dependencies and using multi-tasking on predicting LDA topic distribution. Phase 2. Training ensemble scoring model, we compare the rank of spectra prediction from GNN and MLP encoding on candidate ranking problem for query spectra in the training set to predict a score on weighing the two models. Note that ESP focus on solving the three subproblems of metabolite annotation: GNN and MLP models in Phase 1 focus on (1) molecular encoding; label-mixing and multi-tasking on LDA topic distribution prediction in Phase 1 focus on (2) spectra encoding; ensemble scoring based on ranks of target molecule from two models in Phase 2 focus on (3) candidate ranking.

The ESP model improves on current MLP and GNN models by capturing spectral peak dependencies and by combining spectral predictions of the GNN- and MLP-based models to achieve the highest average rank. The ESP model is trained in two phases (Fig. 2). In Phase 1, GNN- and MLP-based models are trained to predict the spectra. In Phase 2, the spectra prediction models are evaluated on candidate ranking, and the ranking results are used to train an ensemble classifier to judiciously weight the MLP and GNN spectra predictions. Phase 1 thus addresses the first two subproblems and Phase 2 addresses the third subproblem in Fig. 1.

- **Phase 1. Molecular encoding and spectra prediction.** MLP- and GNN-based models are trained in end-to-end fashion to encode the molecules and predict the spectra. Both models utilize the cosine similarity as a loss function. As input, the MLP model utilizes the ECFP molecular fingerprint while the GNN model utilizes the molecular graph. Both models predict the intensity of the spectra binned at 1 Da intervals. To improve over prior GNN (Zhu *et al.* 2020) spectra prediction models, we test various GNN models (Lei *et al.* 2017, Velickovic *et al.* 2017, Schlichtkrull *et al.* 2018, Xu *et al.* 2018) and select the best performing model, Graph Isomorphism Network with Edge features (GINE) (Hu *et al.* 2019). Bidirectional prediction, originally proposed for MLP models (Wei *et al.* 2019), is added to the GNN models to aid in predicting intensities of larger fragments.
  Spectra prediction accuracy is enhanced by incorporating peak dependencies using label-mixing mechanism and spectral topic distributions (motifs). Label-Mixing is applied to capture pairwise peak dependencies. Through multi-task learning, ESP predicts spectral motifs that are learned on our dataset through topic modeling (Blei *et al.* 2003, Wallach 2006). As in prior works (van Der Hooft *et al.* 2016), peaks are assumed words, and spectra are assumed documents. Each spectral topic is modeled as a probability distribution over a vocabulary of peaks [mass-to-charge (*m*/*z*) values and their intensities], while each spectra is modeled as a probability distribution over topics. The latter is referred to as a spectral motif. The MLP and GNN models combined with peak dependencies (PD) are referred to as MLP-PD and GNN-PD, respectively. Further, experimental instrument settings are used in MLP- and GNN-based models, thus explicitly accounting for collision energies and instrument types, and allowing for spectra predicting and candidate ranking under a wide range of instrument settings.
- **Phase 2. Ensemble model training based on candidate ranking.** We train an ensemble model that judiciously combines spectra predicted by the trained GNN-PD and MLP-PD models. Because we aim to solve the candidate ranking problem, the ensemble model is trained to leverage the ranking capabilities of the two models. As the ranking task requires candidate molecules/spectra, our training dataset is stratified by molecular formula, thus forming molecular candidate lists with known spectra. Each paired spectrum/molecule therefore utilizes spectra/molecules within its strata as candidates. As candidates from molecular databases typically do not have known spectra (if they did, we would have used them for training!), this stratification strategy allows us a molecular candidate dataset with known spectra.
  The MLP-PD and GNN-PD are used to rank the candidates. Ranking performance is reported using spectral similarity and the rank of the target molecule within the candidate set. Each training example is thus assigned a ranking under each model. Additionally each example is assigned a label to indicate which of the two models, GNN-PD or MLP-PD, outperforms the other in terms of ranking. Based on the ranking positions and their differences, a score is assigned to each example to indicate its importance in distinguishing between the ranking performance of the two models. The importance score is computed using Symmetric Mean Absolute Percentage Error (SMAPE) and provides higher weights for lower ranks while favoring training examples with better rank performances. Using the importance weights, a classifier, referred to as the *ensemble classifier*, is then trained on the GNN/MLP labels. During test, the predicted label probabilities for a candidate spectra are used to weight the MLP-PD and GNN-PD predicted spectra.

The ESP model is trained on the NIST-20 LC-MS/MS spectra data under positive mode with precursor type [M+H]+, with different collision energy levels (Phinney *et al.* 2013). Spectra is binned into 1000 bins, each covering a 1 Da range of mass-to-charge (*m*/*z*) ratios. To avoid over representing the molecules with numerous spectra under different collision energy levels, a maximum of 5 representative spectra are randomly selected per molecule with replacement. We utilized 89 405 spectra, involving 17 881 molecules. To ensure that the molecules in the test set are not in the training and validation sets, we split the data on molecules. The data was split randomly for training, validation, and test sets at the ratio of 8:1:1. The test set had 12 375 spectra that correspond to 2475 molecules.

During test, molecular candidates are retrieved from PubChem (Kim *et al.* 2016) for each test molecule, where candidates have the same molecular formula as the target molecule. We assume that the target molecule is within the candidate set. To provide consistent comparisons of our results across different models, candidate sets are sampled to allow for a fixed average size of molecules per candidate set. The default average size is 100 molecules, unless noted otherwise. ESP is then applied to measured spectra in the test set and its corresponding candidate set. The ESP model first predicts the spectra under the MLP-PD and GNN-PD models. Then, it combines the spectra based on the probabilities predicted by ensemble classifier on the measured spectra. This last step determines the relative weighting of the MLP-PD and GNN-PD spectral predictions. The ESP model then recommends the molecular candidate whose predicted spectra has the highest spectral similarity to the measured spectra. We report our results by comparing against the baseline MLP (Wei *et al.* 2019) and GNN (Zhu *et al.* 2020) models. Further, the data split during training/testing can be detrimental to performance. In prior work (Dührkop *et al.* 2015), it was demonstrated that splitting training and test sets based on spectra (instead of molecules) can lead to significantly higher performance due to the same molecule appearing in both training and test sets. Here, we demonstrate data split issues when training on well-known molecules and testing on lesser known molecules. In addition, we show that ranking results are dependent on the size and composition of the candidate sets. We train ESP on the NPLIB1 dataset to compare to a similarly trained MIST model(Goldman *et al.* 2023).

### 3.2 ESP outperforms MLP- and GNN-based models based on average rank

To compare the performance of the ESP, MLP- and GNN-based models on the NIST20 dataset, we report the average rank of the target molecule within the candidate set, and the average rank@k with k=1,3,10. The rank@k metric reflects the likelihood of identifying the target molecule among the *k*-top ranked candidates, and the rank@k results are averaged across the test set. ESP outperforms the baseline GNN and MLP models in terms of average rank, as well as the two models augmented with the peak dependency analysis, MLP-PD, and GNN-PD (Table 1). The average rank performance of 213.597 is a significant improvement over the baseline MLP and GNN models. Augmenting the MLP and GNN baseline models with peak dependencies improves the rank@1,3, and 10 performance for both models but the average rank only for the GNN baseline model. The decrease in average rank is due to ESP’s ability to improve the ranking for difficult-to-rank molecules in the dataset (Supplementary Fig. S2).

**Table 1.** Metabolite annotation evaluation on [M+H]+ precursor.^a^

	Average rank	Rank@1	Rank@3	Rank@10	Cosine similarity
			
	The lower the better	The higher the better			The higher the better
A. Training on NIST-20; test candidates from PubChem					
MLP	279.964	0.197	0.303	0.453	0.735
GNN	340.306	0.056	0.137	0.291	0.699
MLP-PD	299.007	**0.205**	**0.318**	0.463	0.733
GNN-PD	320.726	0.075	0.170	0.333	0.694
ESP-SL	275.182	0.190	0.312	0.473	0.738
ESP-RU	214.226	0.167	0.301	0.482	**0.750**
ESP	**213.597**	0.169	0.301	**0.488**	**0.750**
B. Training on NIST-20; test candidates from COCONUT					
MLP-PD	6.653	0.531	0.740	0.877	0.731
GNN-PD	6.339	0.488	0.693	0.859	0.689
ESP	**5.659**	**0.551**	**0.763**	**0.900**	**0.746**
C. Training on NIST-20; test on most similar candidates from PubChem					
MLP-PD	14.333	**0.283**	**0.435**	0.646	0.733
GNN-PD	17.823	0.120	0.256	0.521	0.694
ESP	**13.491**	0.225	0.397	**0.655**	**0.750**
D. Training on NPLIB1; test candidates from PubChem					
MLP-PD	313.927	**0.237**	**0.389**	**0.519**	**0.633**
GNN-PD	**225.928**	0.121	0.270	0.459	0.618
ESP	291.104	0.178	0.331	0.479	0.624

a Performance is presented based on the data on which the models are trained and the source of test candidates. The metrics include average rank, rank@1, 3, and 10, and cosine similarity. Average rank reports on the overall performance of the relevant test set. Rank@k represents the portion of correct identifications when considering the top *k* candidates. Cosine similarity is the average cosine similarity of the predicted test spectra against the ground truth spectra. Models are trained and evaluated on the NIST-20 in all cases except (D), where they are trained on the NPLIB1 dataset. Test candidates include the full candidate set from PubChem in (A), the full candidate set from COCONUT in (B), the 100 most similar candidates from PubChem in (C), and the full candidate set from PubChem in (D). The bold entries include the best rank within each subtable.

**Figure 3.**
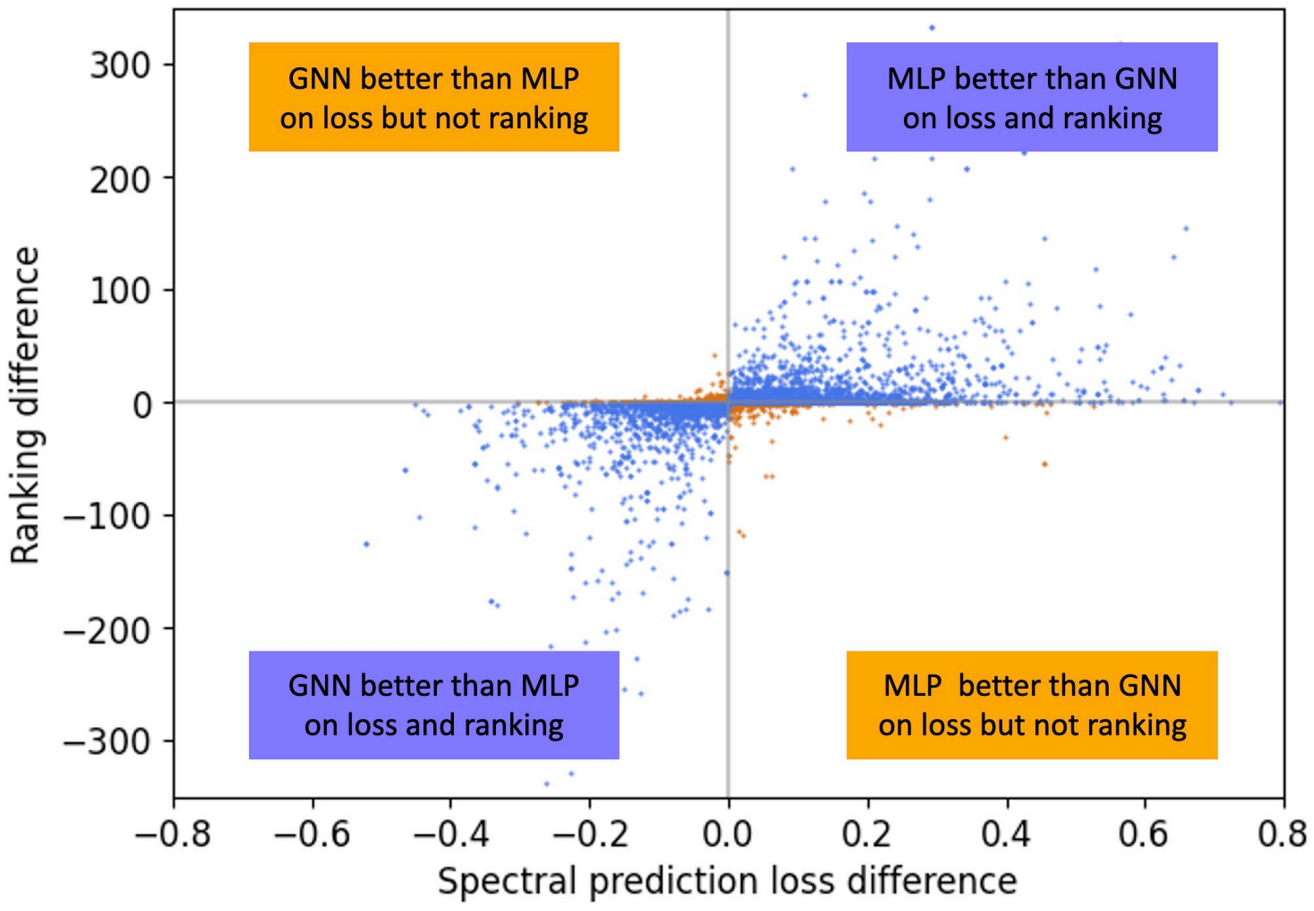
Spectral loss difference (*x*-axis) versus rank difference (*y*-axis) between MLP-PD and GNN-PD results for test set molecules. Spectral prediction loss is computed using the negative cosine similarity between measured and predicted spectra. Candidate ranking performance is the rank of target molecule per each model. The rank and loss difference metrics are in agreement in some cases (points in the upper right and lower left quadrants), but there are cases of disagreement (points in upper left and lower right quadrants).

Preliminary analysis of the GNN and MLP model performance on test sets spurred the idea of combining the two models. For our test set, GNN ranks the target molecule higher than MLP for 24% of the target molecules, while MLP ranks higher on 32% of target molecules. GNN and MLP tie for 44% of the cases. To further understand performance differences, we plot the correlation between rank differences of the MLP-PD and GNN-PD models and their respective spectral loss between measured and predicted spectra for the test set (Fig. 3). In most cases, the spectral prediction loss and ranking differences are in agreement for the two models (blue points in the upper right and lower left quadrants). It is tempting to conclude that spectral prediction loss is a good proxy for the desired ranking tasks. However, there is little, if any, correlation between the spectral loss and the ranking. Therefore, the spectral prediction loss may not strongly predict candidate ranking. As the goal of annotation is to achieve the best possible ranking performance, combining the MLP and GNN spectral outputs based on the ranking results (and not on the spectral loss differences) is a natural next step, and allows benefiting from both GNN- and MLP-based spectra prediction models.

### 3.3 Training on rankings of MLP- and GNN-based models benefits ESP

ESP is trained on the rankings obtained from MLP-PD and GNN-PD. Further, each example is weighted by SMAPE. We first assess the benefits of using ranking instead of spectral loss. We develop a model, ESP-spectra_loss (ESP-SL), where the ensemble classifier is trained using importance weights in proportion to the spectral loss differences (not rank differences). Specifically, the ensemble classifier is trained on the GNN/MLP labels now generated based on the smaller of the two predicted spectra losses. ESP-SL achieves a 275.182 average rank, thus performing better than MLP-PD and GNN-PD models. Next, to evaluate the benefit of weighting each sample via SMAPE instead of uniform weighting, we develop a model ESP-rank_uniform (ESP-RU), where the ensemble classifier is trained on the GNN/MLP labels generated based on rank results, but each training example is weighted uniformly. ESP-RU underperforms ESP or performs equally across all metrics.

### 3.4 Candidate quantity and quality dictate ranking performance

We utilized a fixed average candidate set size of 100 molecules to facilitate our model evaluation efforts. However, the presence of difficult-to-rank molecules even within a fixed average candidate set size led us to analyze and explore candidate molecular sets retrieved from PubChem. Candidate set sizes varied tremendously, where some chemical formula retrieval yielded over 10 000 molecular candidates (Supplementary Fig. S3A). Further, pairwise (MACCS) fingerprint similarity analysis between each test molecule and its candidates revealed a wide range of similarities (Supplementary Fig. S3B). Our hypothesis is that larger candidate sets and those with many similar candidates to the test molecule are more challenging than their counterparts. We explore this hypothesis by varying the size and diversity of the candidate sets for molecules in our test sets.

**Figure 4.**
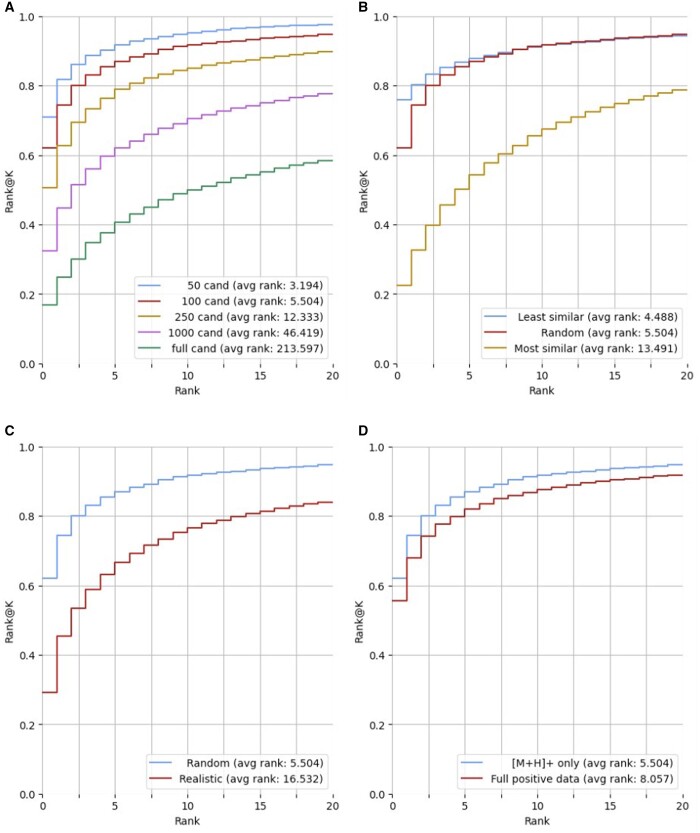
Candidate ranking performances (rank@k) for various settings. Results are reported for a candidate set of 100, unless otherwise noted. (A) Different number of candidates (cand) (50, 100, 250, 1000, full). (B) Least and most similar 100 candidates. (C) Random data split versus realistic split assuming 100 candidates. (D) ESP performance on full positive dataset versus ESP performance on precursor [M+H]+ only.

The ESP model was used to predict and rank spectra for the same test target molecules but with different number of candidates molecules (Fig. 4A). The candidate sets with differing sizes were generated by randomly sampling from the candidates retrieved from PubChem. We vary the candidates size to 50, 100, 250, and 1000 molecules and report on rank@k, for *k *=* *1 to *k *=* *20. In all cases, the target molecule is included in the candidate set. Clearly, smaller candidate sizes yield better candidate ranking, concluding that the size of the candidate set has a significant impact on candidate ranking performance. The same trend holds for the MLP-PD and GNN-PD models (Supplementary Fig. S4A–D).

To assess the difficulty in ranking due to the similarity of the candidate molecules to the target molecule, two 100-molecule candidate sets generated for each target test molecule: those that are most and least similar to the test molecules. Molecular similarity is calculated using pairwise MACCS fingerprint between candidate and target molecules. ESP performs best on the least similar candidates (Fig. 4B) and shows higher rank performance until rank 8, after which the model has comparable rank@k performance with randomly selected candidate sets. As expected, the model is more challenged with sets of candidates most similar to the target molecule than when using a randomly selected set of candidates (Fig. 4B). The rank@k performance decreases for all shown ranks. Importantly, a high similarity yet smaller candidate set (100 molecules) results in poorer performance when compared to a large (1000 molecules) randomly selected candidate set, up to the rank of 17. The performance of MLP-PD and GNN-PD follow a similar trend to ESP (Supplementary Fig. S5A and B). Further, MLP-PD outperforms ESP for rank 1–8 on the most similar dataset, highlighting the value of domain expertise in the fixed fingerprints and gaps in GNN representation learning.

### 3.5 Realistic data split

Another source of difficulty in ranking candidates is the lack of similar molecules or spectra in the training dataset. While some molecules are popular in databases due to their known biological or chemical significance, such as their role in primary metabolism or their environmental relevance, there is limited documentation for many metabolic products. We evaluate how the model performs under such a “realistic split” scenario (Martin *et al.* 2017), where the model is trained on well annotated molecules but tested on less popular molecules. Molecules are clustered based on MACCS molecular fingerprint similarity. UPGMA (the unweighted pair group method with arithmetic mean) (Sokal 1958) clustering method is applied on the first two dimension of t-SNE for the molecular fingerprint space (Supplementary Fig. S6A). This clustering method results in 50 generated clusters. The larger 29 clusters were used for training, while the smaller 21 clusters were used for testing. This specific split was selected to ensure models trained on realistic split are exposed to similar number of training data points as used in random split. ESP ranking under the realistic split drops when compared to the performance on a random split (Fig. 4C), but the gap under the two splits narrows at higher ranks. This drop in performance is consistent for the MLP-PD and GNN-PD models (Supplementary Fig. S6B). The performance of the GNN-PD is a close match to that of ESP, highlighting again the importance of learned representations.

### 3.6 Performance on the NPLIB1 dataset

To compare with other tools, we benchmark the performance of ESP against MIST (Goldman *et al.* 2023), the current best ranking tool. The MIST model is trained on the publicly available NPLIB1 dataset released with the CANOPUS tool (Dührkop *et al.* 2021), after filtering it to include only [M+H]+ spectra. This dataset is culled from reference MS/MS libraries, and it includes 8030 spectra from 7131 unique compounds. Since collision energies were not part of the NPLIB1 dataset, we exclude such information in our model. The models are trained on the same data split as the one used in MIST. The performance evaluation on the NPLIB1 dataset (Table 1D) shows that GNN-PD achieves the lowest rank when compared to MLP-PD and ESP. However, MLP-PD consistently outperformed GNN-PD and ESP at ranks 1 through 20. Unlike the evaluation on the NIST dataset (Table 1A–C), ESP does not achieve the best average rank. We conjecture that the underlying data distributions are different between the NPLIB1 and NIST-20 datasets.

Training on the NPLIB1 dataset resulted in a decrease in cosine similarity (0.750 versus 0.624 for NIST-20 versus NPLIB1). On the same dataset, MIST achieved a cosine similarity between 0.675 and 0.700, per Figure 6a in reference (Goldman *et al.* 2023) and rank@1 of 0.279, rank@3 of 0.533 and rank@10 of 0.719. The rank@k values were obtained by rerunning MIST while trained on the NPLIB1 dataset. The MIST results were confirmed with the lead MIST author. ESP therefore does not outperform MIST in terms of cosine similarity nor in top rank, as MIST utilizes domain knowledge, such as precursor formulas and peak annotations, while ESP utilizes molecular fingerprints and structure.

### 3.7 Full positive mode model performance

Provided with the appropriate data, the ESP model can be trained to predict spectra under various instrument settings, including precursor types and collision energies. To evaluate ESP’s performance on multiple instrument setting, we train and replicate the experiments on all positive mode spectra from the NIST20 dataset with various precursor types such as [M+H]+, [M+H-H2O]+, [M+H-2H2O]+, [M+H-NH3]+, and [M+Na]+. The relative performance of the ESP models on all positive mode spectra is consistent with the performance of ESP on only [M+H]+ precursor types with some degradation (Fig. 4D). The performance trend is similar for the GNN-PD and MLP-PD models trained under multiple instrument settings (Supplementary Fig. S6C and Supplementary Table S1).

## 4 Discussion

This work proposed a novel molecule-to-spectra prediction model, ESP, for metabolite annotation. The model learns a weighting on the outputs of MLP and GNN spectra predictors. The average rank performance of the MLP and GNN models are enhanced by peak dependency considerations, including the addition of label-mixing mechanism on peaks within the spectra and multi-tasking on spectral topics. The ensemble model significantly improves average candidate ranking performance over MLP and GNN baseline models.

An important aspect of this work is addressing challenges in training and test strategies in terms of (i) data splits, (ii) size and quality of candidate datasets, and (iii) training datasets. While it is common from a machine learning perspective to stratify the datasets such that the test data distribution mirrors the training data (de Jonge *et al.* 2022), we designed a data split (realistic split) to highlight the challenges facing annotation tools where the target molecule is novel, and not as well representative within the training set as other molecules. The average rank increases from 5.504 to 16.532 when considering 100 random molecules from the candidate set (Fig. 4C). In regards to the candidate datasets, this work demonstrated the strong dependence of the results on the candidate set size (average rank changes from 3.194 for 50-molecule candidate size to 213.597 for the full candidate set) (Fig. 4A). Further, there is strong dependence on the candidate similarity to the target molecule (average rank changes from 4.488 for least similar candidates to 13.491 for most similar candidates) (Fig. 4B). These drops in performance were observed across all our machine learning models, and we expect this trend to persist across other machine-learning based models. The evaluation results on the NPLIB1 dataset differed from those on NIST-20 (Table 1D), where GNN-PD was best for average rank and MLP-PD is best for rank@k and cosine similarity. We suspect that the emergence of deep-learning annotation models will unify our community efforts in benchmark development beyond the NPLIB1 dataset.

ESP innovates over prior molecule-to-spectrum prediction approaches and provides several insights. First, ESP is designed to exploit both molecular fingerprints and graph structure. Our own prior work (Zhu *et al.* 2020) showed that MLPs outperform or are on par with Graph Attention Networks (GATs) (Veličković *et al.* 2017) when predicting the top rank. This result stands even when pre-training GNNs on a large molecular dataset (Young *et al.* 2021). Our experiments herein showed that the MLP-PD model was more effective at rank@1 for all scenarios except for the COCONUT dataset evaluation (Table 1B). ESP excelled at reducing the average rank for all scenarios, except when training and testing on the NPLIB1 dataset. GNN-PD seemed to rarely outperform ESP and MLP-PD models, except on the average rank for the NPLIB1 dataset. Second, ESP demonstrated two successful yet complementary strategies: label-mixing mechanism on the peaks, and learning spectra motifs as secondary task via multi-task learning. Third, our analysis provided the insight that spectral loss weakly correlates with rank and we showed that spectral loss is less competitive when compared to ranking loss. Deep learning models thus will benefit from rank-learning tasks instead of relying on spectral loss, a common practice. Fourth, while we performed model selection during training on the basis of average rank, we could alternatively select the model with the best top rank, or rank@k for a specified *k*. Focusing on average rank allowed us to research the presence and rationale for “difficult-to-rank” molecules. Finally, ESP stratifies the training data by molecular formulas to create candidate sets, thus avoiding the retrieval of candidate molecular sets as in prior works (Brouard *et al.* 2017). This strategy proved successful in training ESP on a ranking task. The broader idea, however, is that stratifying training data can maximize the challenging task of within-strata discriminative learning.

When trained on the NPLIB1 dataset and compared against the MIST model, which was also trained and evaluated on the same dataset, ESP unperformed MIST in terms of cosine similarity and rank@k. While ESP is a molecule-to-spectrum approach that utilizes additional information in the form of spectral topics to learn peak dependencies, MIST is spectrum-to-molecule approach that utilizes chemical formulas for the peaks. Both works highlight the need to incorporate additional data to arrive at higher performance. Prior works have augmented candidates with additional information to enhance ranking predictions. MetFrag utilizes additional information such as citations to prioritize the candidates (Ruttkies *et al.* 2016). CSI:FingerID utilizes fragmentation trees that best explain the spectra (Rauf *et al.* 2013, Dührkop and Böcker 2015) to improve mapping candidates to their corresponding fingerprints (Dührkop *et al.* 2015, Vaniya and Fiehn 2015). In addition to learning to rank, we expect auxiliary information in various forms, such as biochemical (Shen *et al.* 2019) data or data augmentation (Shrivastava *et al.* 2021) to improve annotation.

ESP was trained assuming that the target molecule is in the candidate set. However, that is not always the case, especially when utilizing smaller datasets such as the COCONUT dataset. Our current training methodology does not account for this case. Ideally, the training procedure should include a loss function to maximize the likelihood of matching on the candidate with the highest molecular similarity to the target molecule. An alternative approach is to use companion approaches such as MS2Query (de Jonge *et al.* 2023) MS2Query or MS2Deepscore (Huber *et al.* 2021b) for analogue searches. Devising strategies to engineer an appropriate candidate set (Hassanpour *et al.* 2020) or to constraint *de novo* molecular generation becomes an important aspect of improving annotation.

Another important aspect of this work is showing that neural networks can be adapted to learn spectral prediction under different collision energies and adducts. However, as demonstrated, there is a drop in performance when training/evaluating for a wide range of instrument settings. Deep learning techniques such as domain generalization and zero-shot learning stand to further enhance this aspect of metabolite annotation.

The ESP model predicts the spectra for a given molecule. To use ESP to annotate a measured query spectrum, the user can supply a set of candidate molecules for each measured spectra. Such a set can be retrieved from PubChem or other biologically relevant molecular databases based on putative chemical formulae or molecular mass (+/- some threshold). BUDDY, for example, achieves 93.0% accuracy on for *m*/*z* < 400 (Xing *et al.* 2023). The user can then use the cosine similarity to evaluate the similarity between the query and predicted spectra to rank the candidate set to report the ranking, as we performed when evaluating ESP. As we demonstrated, the quality and quantity of the candidate set size has a profound impact on the results. Hence, candidate set design, e.g. identifying biologically relevant candidate molecules using biotransformation rules (Hassanpour *et al.* 2020) or generating chemically relevant *de novo* candidate molecular structures, can significantly aid in improving annotation quality.

Our ESP model and others are trained and evaluated on reference mass spectral libraries [e.g. the well-curated proprietary NIST and METLIN (Smith *et al.* 2005) as well as public libraries including MassBank (Horai *et al.* 2010), GNPS (Wang *et al.* 2016), MoNA, and others], typically utilized for spectra library search. These models are selected because they are readily available through purchase or in the public domain. As typical in supervised Machine Learning applications, the performance is reported on various data splits from the same dataset. However, models trained on reference dataset may not generalize well to noisy experimental spectra. Therefore, there remains a need to train and evaluate ML-models on noisy spectral datasets.

## Supplementary Material

btae490_Supplementary_Data
