# Supplementary material for: An Ensemble Spectral Prediction (ESP) model for metabolite annotation
Source: Bioinformatics. 2024 Aug 17;40(8):btae490. doi: 10.1093/bioinformatics/btae490 (PMC11344591; doi:10.1093/bioinformatics/btae490)
Supplement: btae490_Supplementary_Data [file btae490_supplementary_data.pdf]

Ensemble Spectra Prediction (ESP) model for metabolite annotation

Xinmeng Li, Yan Zhou Chen, Apurva Kalia, Hao Zhu, Li-Ping Liu, and Soha Hassoun
Department of Computer Science
Tufts University, Medford, MA 02155

Supplementary File

S1 Conceptual framework for solving the spectrum-to-molecule approach

A conceptual framework for solving the three subproblems involved in the spectrum-to-molecule annotation problem (Fig. S1).

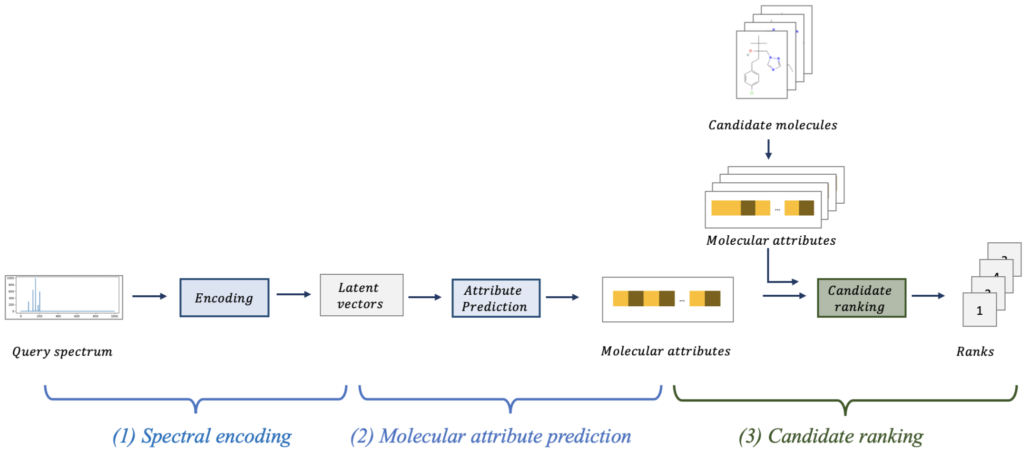

Figure S1: A conceptual framework for solving the three subproblems involved in the spectrum-to-molecule annotation problem: (1) representation learning of query spectra, (2) molecular attribute prediction from spectral representation, and (3) ranking of candidate molecular attributes against predicted attributes. Shown is a prediction of molecular attributes; however, the ranking is still applicable when predicting candidate de novo molecular structures.

S2 The Presence of difficult-to-rank molecules

We plot the distribution of the molecules at each rank (Fig. S2). All models do well in predicting the target molecule correctly for a great number of molecules, and thus the high number of molecules at small rank values. However, all models are challenged by difficult-to-rank molecules that result in high rank. These molecules directly impact the average rank.

Our work herein in terms of peak dependency considerations and the learning on rank address this challenge. Both MLP and GNN models improve in this regard when including peak dependencies (Fig. S2A,B). The performance of ESP on the difficult-to-rank molecules is also improved when compared to the ESP-SL and the ESP-RU models (Fig. S2C,D), thus supporting the improvement in average rank for ESP over these two models.

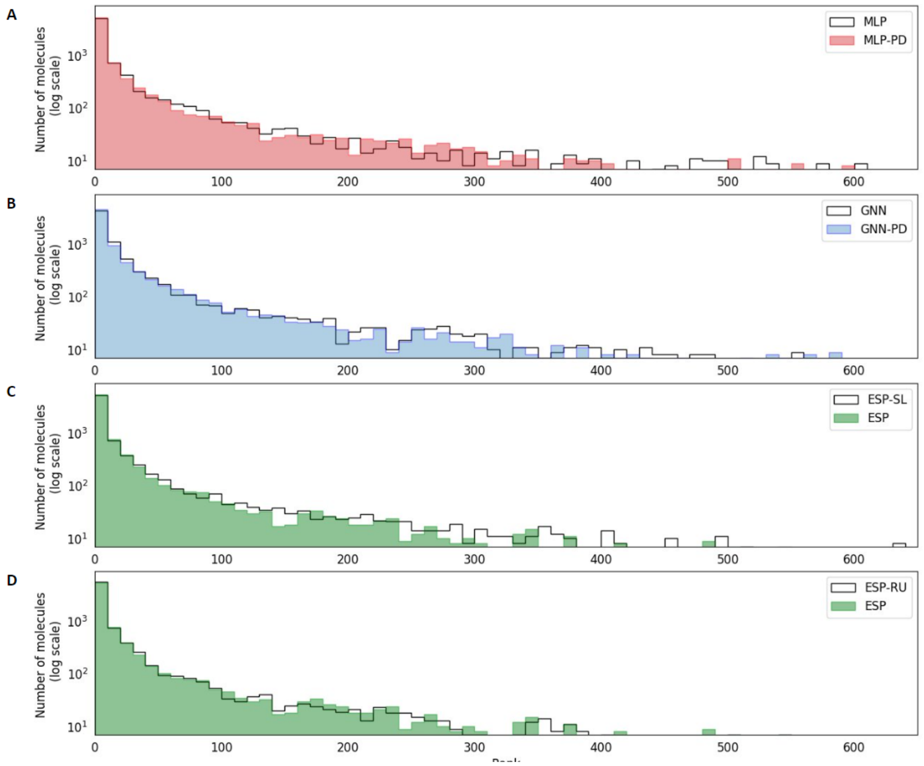

Figure S2: Number of test molecules at a particular rank. Our model improvements address the difficult-to-rank molecules and hence improve the average rank. A) MLP-PD vs MLP model. B)GNN-PD vs GNN model. C) ESP vs ESP-SL model D) ESP vs ESP-RU model.

Table S1: Ablation study on the ESP model. See text on ablation studies in this supplement for experimental details. The evaluation is on [M+H] precursor mode data for 100-molecule average candidate set size under random data split. Average rank reports on the overall performance on the test set. Rank@k represents the portion of correct identifications when considering the top k candidates.

	Average rank	Rank@1	Rank@3	Rank@10
	The lower the better	The higher the better		
MLP	6.935	0.584	0.756	0.879
GNN	8.246	0.472	0.719	0.865
MLP-LDA	6.924	0.589	0.764	0.880
GNN-LDA	7.760	0.499	0.726	0.870
MLP-MixL	7.308	0.585	0.759	0.877
GNN-MixL	8.107	0.482	0.728	0.870
MLP-PD	7.272	0.592	0.756	0.878
GNN-PD	7.817	0.507	0.730	0.870
CONCAT-ENS	7.816	0.506	0.730	0.871
AVG-ENS	8.997	0.465	0.691	0.846
ESP-SL	6.764	0.600	0.763	0.884
ESP-RU	**5.491**	0.619	**0.799**	0.911
ESP	5.504	**0.620**	**0.799**	**0.912**

S3 Ablation studies on the ESP Model

To provide further detailed evaluation on the ESP model components, we evaluate the following variations of the model and report the results in Table S1.

- MLP - Baseline
- GNN - Baseline
- MLP-MixL - MLP + label-mixing layer
- GNN-MixL - GNN + label-mixing layer
- MLP-LDA - MLP + multitasking on spectral motifs
- GNN-LDA - GNN + multitasking on spectral motifs
- MLP-PD - MLP + label-mixing layer + multitasking on spectral motifs
- GNN-PD - GNN + label-mixing layer + multitasking on spectral motifs
- CONCAT-ENS - Ensemble model using the concatenation of MLP and GNN embedding
- AVG-ENS - Ensemble model taking the average of MLP and GNN spectral predictions, thus weighting each equally of the predicted spectra equally
- ESP-SL - Ensemble classifier is trained using importance weights in proportion to the spectral loss differences (not rank differences).
- ESP-RU - Ensemble classifier is trained on the GNN/MLP labels generated based on ranking results, but each training example is weighted uniformly.

Based on these results, the ESP model is the best performing model in every category except average rank, where the performance is marginally worse than ESP-RU.

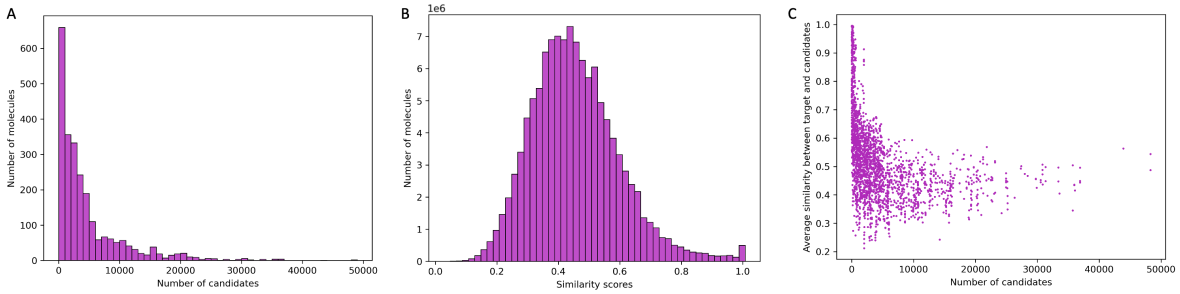

Figure S3: Profiling candidate molecules retrieved from PubChem. A) Histogram of number of candidates (x-axis) for the test molecules. B) Histogram of pairwise MACCS fingerprint similarity between target molecules and their respective candidates. C) Scatter plot of candidate sets showing the size of the candidate-set (x-axis) against similarity between target and candidates in each candidate set.

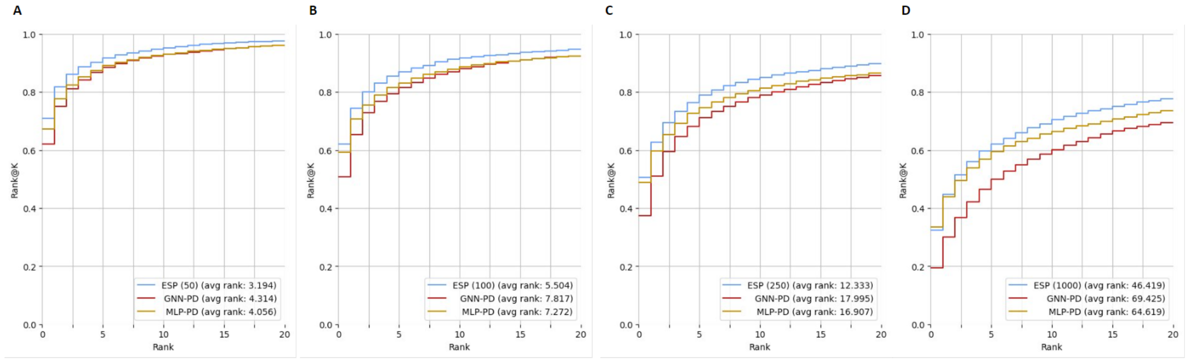

Figure S4: Comparing rank@k performance for ESP, GNN-PD, MLP-PD models for different candidate set sizes of: A) 50 molecules, B) 100 molecules, C) 250 molecules, D) 1,000 molecules.

S4 Candidate set distributions

Distributions on the number of candidate sets retrieved from PubChem for each molecule in our test set show a long tail distribution (Fig. S3A). The average number of candidates was 4,728 with a maximum of 48,292 candidates. The similarity of the candidate sets to the target molecule shows a normal distribution (Fig. S3B). The sets with high molecular similarities indicate that there are candidates that are difficult to rank. Our experiments show that ESP (and other models) are more challenged by high similarity candidate sets. There is weak correlation between the similarity and size of the candidate sets (R^2 is -0.63) (Fig. S3C).

S5 ESP, MLP-PD and GNN-PD performance as a function of candidate set size and similarity

Both MLP-PD and GNN-PD models are more challenged with increasing dataset sizes from 50, to 100 to 250 to 1,000 candidates (Fig. S4). ESP outperforms GNN-PD and MLP-PD on the least similar candidate sets (Fig. S5A). However, on the most similar candidate set, MLP-PD outperforms ESP on ranks 1-3, but ESP outperforms MLP-PD on higher ranks (Fig. S5B). See also Table 1(B) in the main text.

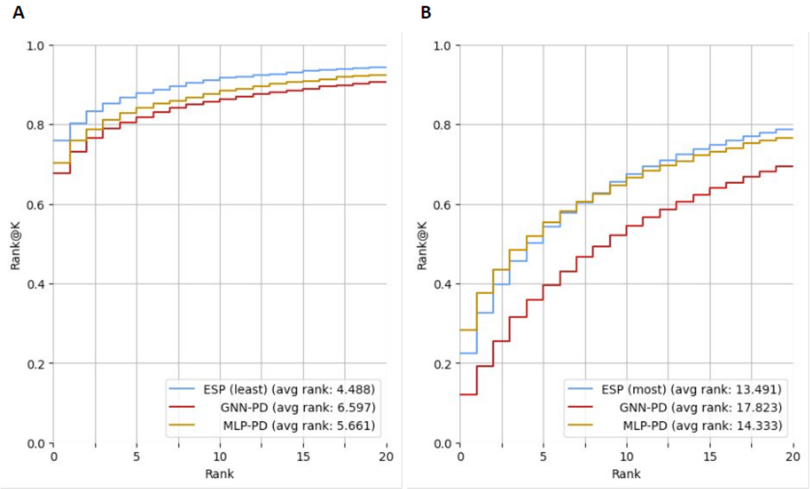

Figure S5: Comparing rank@k performance for ESP, GNN-PD, MLP-PD models for different candidate sets: A) least similar candidate sets, B) most similar candidate sets.

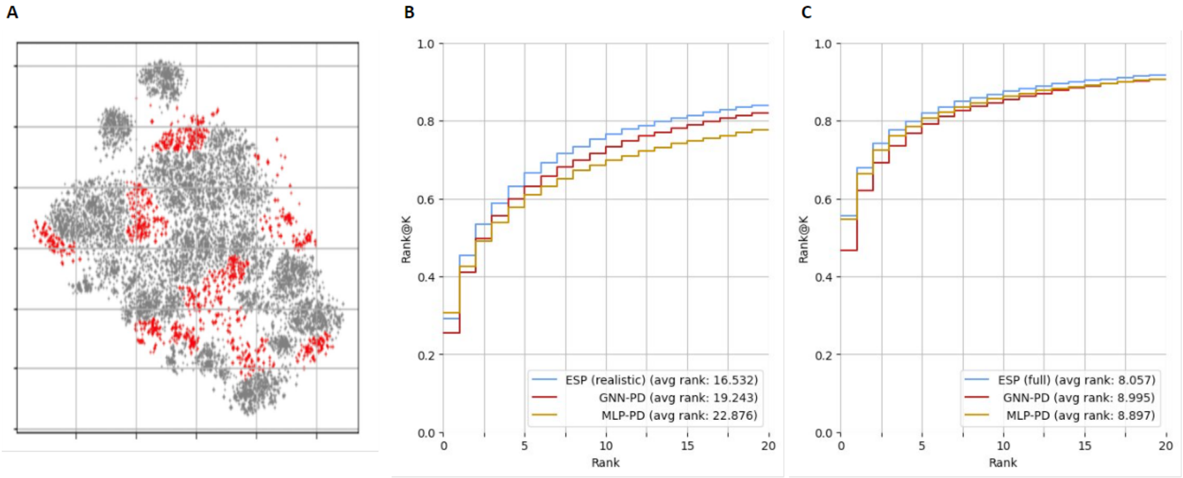

Figure S6: Analysis for realistic data splits. A) Realistic split t-SNE plot, where grey clusters are. used for training and red clusters are used for test). B) Comparing rank@k performance for ESP, GNN-PD, MLP-PD models under realistic split set. C) Comparing rank@k for the full positive ion data set.

Table S2: Metabolite annotation evaluation on full positive ion mode.

	Average rank	Rank@1	Rank@3	Rank@10
	The lower the better	The higher the better		
MLP	8.584	**0.562**	0.728	0.853
GNN	9.536	0.468	0.686	0.844
MLP-PD	8.897	0.547	0.725	0.856
GNN-PD	8.995	0.466	0.692	0.846
ESP-SL	8.507	0.550	0.732	0.861
ESP-RU	8.201	0.553	0.736	0.865
ESP	**8.057**	0.556	**0.741**	**0.867**

S6 MLP-PD and GNN-PD performance on realistic data splits and full-positive mode

The t-SNE plot shows distinct clusters on the test molecules (Fig. S6A). Under the realistic split, the models are trained on the larger clusters and tested on the smaller clusters. ESP marginally outperforms GNN-PD under the realistic split (Fig. S6B). When training on the full positive mode dataset, performance drops for all models (Table S2, Fig. S6C).

S7 Annotation example

We provide an annotation example to highlight the influence of candidate similarity on candidate ranking performances across all models. (Fig. S7). While the three baseline models rank the target molecule among the top 3 candidates, ESP provides the correct ranking for the target molecule. MLP-PD and GNN-PD provide improved ranking over the baselines, but rank the target molecule in second position.

S8 Peak dependency modeling

A label-mixing layer allows modeling peak dependencies (Fig. S8). The spectra with label-mixing, $\hat{\mathbf{y}}_{co}$, is computed based on L label-mixing layers. Label-mixing is learned through a lower dimensional matrix D.

S9 Model tuning

For MLP and GNN baseline models, we follow the author's recommended guidelines on hyperparameter tuning. Otherwise, the range of hyperparameter search is specified as follows. The dimension of the two hidden layers of encoding ECFP fingerprint and instrument setting features are selected from {64, 128, 256, 512, 1024}. For all MLP, GNN, and ensemble classifier training, we optimize our models with the Adam optimizer (Kingma and Ba, 2014) with learning rates selected among $\{10^{-2}, 5*10^{-3}, 10^{-3}, 5*10^{-4}, 10^{-4}, 5*10^{-5},\}$. We apply dropout at a rate selected from $\{0.0, 0.3, 0.5, 0.7\}$ and L2 norm at a weight selected from $\{10^{-2}, 10^{-3}, \ldots, 10^{-6}\}$. For ensemble weighting strategy, we allow a maximum of 200 epochs.

References

Kingma, D. P. and Ba, J. (2014). Adam: A method for stochastic optimization. *arXiv preprint arXiv:1412.6980*.

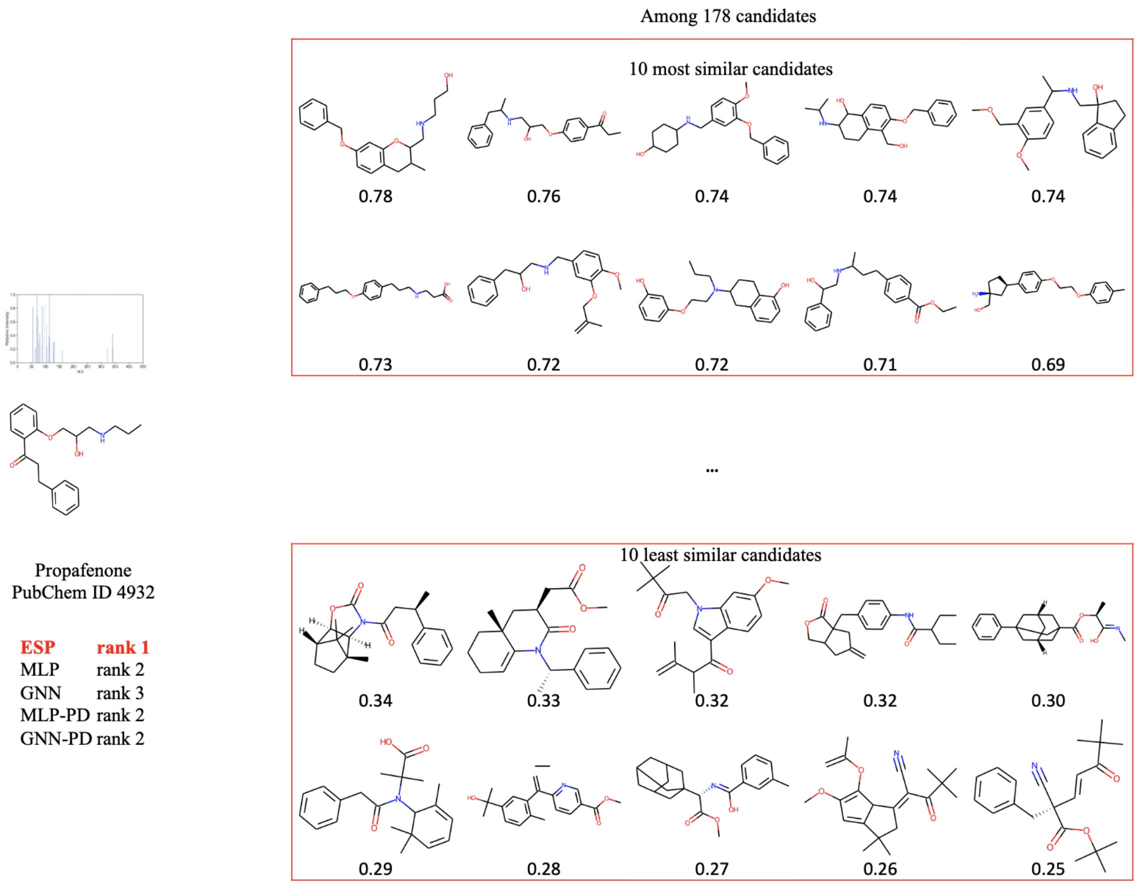

Figure S7: Metabolite annotation example for target molecule Propafenone. Shown are the 10 most and least similar candidates with their respective fingerprint similarity scores.

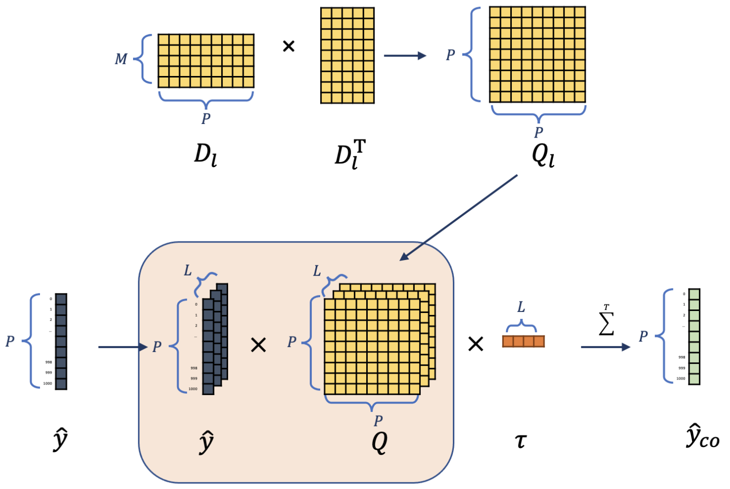

Figure S8: Using label mixing to capture co-occurring spectral peaks. This mixing is applied based on previous prediction $\hat{\mathbf{y}}$, co-occurrence matrix $\mathbf{Q}$, and weight matrix τ. The co-occurrence matrix, $\mathbf{Q}$, is approximated from the learned lower-dimension matrix $\mathbf{D}$.
